# Six weeks stretching of lower limbs lowers blood pressure in hypertensive individuals

**DOI:** 10.1007/s00421-026-06358-8

**Published:** 2026-07-18

**Authors:** Nicholas Toninelli, Alessandro Maloberti, Emiliano Cè, Cristina Giannattasio, Fabio Esposito

**Affiliations:** 1 Department of Biomedical Sciences for Health (SCIBIS), University of Milan, Via Colombo 71, 20133 Milan, Italy; 2 School of Medicine and Surgery, University of Milano-Bicocca, Milan, Italy; 3 Cardiology IV, A. De Gasperis Department, Ospedale Niguarda Ca’ Granda, Milan, Italy

**Keywords:** Arterial stiffness, Vascular responsiveness, Cardiovascular health

## Abstract

**Purpose:**

Previous studies on healthy subjects suggest that 6-week passive stretching (PS) lowers blood pressure (BP) and arterial stiffness by enhancing vascular responsiveness in both stretched and non-stretched arteries. This study investigated whether a similar 6-week PS intervention applied to the lower limbs could elicit similar effects in patients with essential hypertension.

**Methods:**

24 patients underwent assessments of central arterial stiffness (carotid–femoral pulse wave velocity, PWV_cf_), brachial (systemic) and femoral (local) vascular responsiveness (flow-mediated dilation, FMD; nitrate-mediated dilation, NMD; and single passive limb movement, sPLM), as well as BP and heart rate recordings. Measurements were obtained at baseline, after a 6-week control period, and following PS and a follow-up of the same duration.

**Results:**

No changes occurred following the 6-week control period. After PS, systolic, diastolic, and mean BP decreased by 4.9(SD4.6)% (*P* < 0.001, *ES* = –0.99), 6.9(SD4.9)% (*P* < 0.001, *ES* = –1.43), and 3.7(SD6.2)% (*P* = 0.005, *ES* = –0.63), respectively. Peak *Q̇*_*fem*_ increased by 18.4(SD19.0)% (*P* < 0.001, *ES* = 0.92), and Δ *Q̇*_*fem*_ by 27.1(SD21.7)% (*P* < 0.001, *ES* = 0.98). PWV_cf_, FMD, NMD, and heart rate remained unchanged. No correlations were retrieved between percentage differences in *Q̇*_*fem*_ and BP variables. Furthermore, all improvements returned to baseline during follow-up.

**Conclusion:**

These findings indicate that 6-week PS effectively reduces blood pressure in hypertensive patients, possibly due to improved vascular responsiveness in the stretched arteries and the combined effect of other mechanisms that cannot be identified individually. However, these benefits dissipate once the intervention is discontinued.

**Supplementary Information:**

The online version contains supplementary material available at https://doi.org/10.1007/s00421-026-06358-8.

## Introduction

Hypertension is a chronic condition characterized by persistently elevated blood pressure (BP) and an increased risk of cardiovascular events, including stroke, myocardial infarction, heart failure, and premature mortality (Oparil et al. 2018; Gabb 2020). Endothelial dysfunction and arterial stiffness are key mechanisms underlying hypertension development (Dharmashankar and Widlansky 2010; Humphrey et al. 2016). Endothelial dysfunction, defined as impaired arterial vasodilatory capacity, represents an early stage of atherosclerosis (Widlansky et al. 2003; Gimbrone and García-Cardeña 2016). Reduced vascular responsiveness to pressure changes and increased arterial stiffness, driven by decreased elastin and increased collagen content in the arterial wall, contribute to elevated systolic BP (SBP) and diastolic BP (DBP), ultimately increasing cardiovascular risk and mortality (Kim and Kim 2019; Saz-Lara et al. 2022; Kim 2023).

Alongside pharmacological therapy, lifestyle modifications such as regular physical activity are recommended for the management of essential hypertension (Moiz et al. 2024). In this regard, a recent meta-analysis of randomized controlled trials reported that multiple exercise modalities are effective in lowering resting BP. Specifically, significant reductions in resting SBP/DBP were observed following aerobic training (− 4.49/ − 2.53 mmHg), dynamic resistance training (− 4.55/ − 3.04 mmHg), combined training (− 6.04/ − 2.54 mmHg), high-intensity interval training (− 4.08/ − 2.50 mmHg), and isometric exercise training (− 8.24/ − 4.00 mmHg) (Edwards et al. 2023). However, these forms of active exercise may be poorly tolerated by individuals with low exercise tolerance, reduced mobility and/or while bedridden, who are often at greater cardiovascular risk (Lim et al. 1996; Fitzgerald et al. 2015).

Passive static stretching (PS) is a highly tolerable exercise modality used to restore or increase joint range of motion (Bryant et al. 2023). Recent findings suggest that PS interventions can improve vascular responsiveness and reduce arterial stiffness in both healthy individuals and patients with cardiovascular conditions (Kruse and Scheuermann 2017; Hotta et al. 2019; Bisconti et al. 2020; Cè et al. 2022). These adaptations appear to result from repeated exposure to acute PS, during which local and systemic mechanisms modulate vascular function across elongation and recovery phases (Venturelli et al. 2019; Bisconti et al. 2020). During stretching, mechanoreceptor activation increases sympathetic activity and transiently reduces blood flow in unstretched regions, whereas the local release of vasoactive substances increases antegrade blood flow and shear rate in the stretched muscles (Venturelli et al. 2017, 2019; Wong and Figueroa 2021). In subsequent elongation phases, transient reductions in blood flow and tissue oxygenation may create a temporary ischemic–hypoxic environment (Kruse et al. 2016; Hotta et al. 2018). Following stretch release, reactive hyperaemia increases shear rate and endothelial nitric oxide synthase activity, promoting vasodilation (Sandoo et al. 2010). Additionally, PS induces a shift toward greater parasympathetic modulation after the stretching procedure (Wong and Figueroa 2021). Repeated cyclic changes in shear rate and autonomic balance may therefore act as a form of “vascular training”, promoting endothelial remodelling, improved vascular function, reduced arterial stiffness, and angiogenesis even in non-stretched regions (Nishiwaki et al. 2015; Green et al. 2017; Kato et al. 2020; Thomas et al. 2021). These adaptations may ultimately contribute to reductions in BP and improved cardiovascular regulation.

Several studies in hypertensive individuals have reported reductions in BP alongside improvements in vascular responsiveness and sympathetic regulation following full-body PS interventions. Wong and Figueroa (2014) observed reductions in SBP/DBP (~ 5/ ~ 4 mmHg) and improved sympathetic vasomotor activity after 8 weeks of full-body PS in obese postmenopausal women with elevated BP. Similarly, Ko et al. (2020) reported greater BP reductions after 8 weeks of full-body PS compared with brisk walking. Conversely, Yamada et al. (2022) found no significant changes in BP or vascular responsiveness after 6 months of full-body PS, although the small sample size (n = 10) may have limited statistical power. A recent meta-analysis reported significant reductions in SBP/DBP following longitudinal PS interventions (− 5.39/ − 3.93 mmHg) but highlighted substantial heterogeneity and methodological limitations that limit the robustness of the evidence (Themistocleous et al. 2026).

To date, no studies have investigated whether PS can reduce central arterial stiffness and improve vascular responsiveness in both stretched and non-stretched arteries, leading to reductions in BP in hypertensive patients receiving stable antihypertensive medication, using gold-standard assessment techniques. In healthy active young individuals, a 40-min lower-limb PS protocol performed five times per week improved local vascular responsiveness and reduced central arterial stiffness after 6 weeks, with these effects persisting for up to 6 weeks after the intervention (Bisconti et al. 2020). Improvements in BP and systemic vascular responsiveness became evident after 8 (Cè et al. 2022) and 12 weeks (Bisconti et al. 2020) using the same protocol, whereas shorter interventions (4 weeks) appeared insufficient to induce systemic vascular adaptations (Higaki et al. 2022).

Therefore, a 6-week supervised lower-limb PS protocol was selected to investigate the effects of PS on BP, central arterial stiffness, and both local and systemic vascular responsiveness in hypertensive individuals. In addition, follow-up assessments were included to evaluate the persistence of any observed adaptations. We hypothesized that both local and systemic mechanisms would contribute to improved vascular responsiveness in arteries directly and indirectly exposed to the stretching stimulus, leading to reductions in central arterial stiffness and BP, with a potential reversal of these adaptations after cessation of the intervention.

## Methods

### Participants

The study was conceived as a within-subjects longitudinal design with repeated measures. Based on previous research (Bisconti et al. 2020) the required sample size was calculated using changes in flow mediated dilation (FMD) as this variable exhibited the greatest variability. A two-way ANOVA (within-group factor: time; between-group factor: intervention) was performed using an effect size F = 0.255, an alpha level of α = 0.05, and a power of 1-β = 0.80. The analysis was conducted using a statistical software (G-Power 3.1, Düsseldorf, Germany), resulting in 24 participants. Accordingly, 24 participants with essential hypertension were recruited from the Hypertension Clinic of Cardiology 4 Unit at ASST GOM Niguarda Hospital (Milan, Italy). Based on the short-form International Physical Activity Questionnaire (IPAQ-SF), 75.0% of participants were classified as active, 20.8% as moderately active, and 4.2% as inactive. The characteristics of the participants are presented in Table 1. Inclusion criteria were: (i) arterial hypertension for at least 1 year; (ii) treatment with at least two antihypertensive agents (angiotensin-converting enzyme inhibitors, angiotensin receptor blockers, hydrochlorothiazide and thiazide-like diuretics, and calcium channel blockers); and (iii) stable antihypertensive therapy for at least 6 months. Exclusion criteria included: (i) neurological, vascular, or musculoskeletal disorders of the lower or upper limbs; and (ii) changes in medication treatment in the 3 months prior to the intervention and during the study period, including pharmacological treatments affecting neural and/or endothelial responses including beta-blockers or doxazosin and hormonal contraceptives. Additionally, participants could not be involved in any other regular PS program. All procedures were approved by the local ethics committee and complied with the principles of the latest version of the Declaration of Helsinki. All the participants were informed about purpose, design, procedures and possible risks of the study and gave their written informed consent. Participants were informed that they could withdraw from the study at any point without obligation.

**Table 1:** Participants characteristics

Characteristics	Mean(SD)/% N = 24
Male sex (%)	54.2
Age (y)	65.3(8.6)
BMI (kg/m^2^)	28.2(4.7)
Self-reported physical activity levels	
Active [% (MET/day)]	75.0%(0.7)
Moderately active [% (MET/day)]	20.8%(1.7–5.9)
Inactive [% (MET/day)]	4.2%(6.2–57.0)
Years of hypertension (y)	15.2(8.3)
Resistant hypertension	50.0%
Smokers	
Current (%)	4.2
Former (%)	45.8
Creatinine (mg/dl)	0.95(0.28)
CKD (%)	8.3
Glucose (mg/dl)	108.1(18.6)
Total cholesterol (mg/dl)	167.8(35.7)
HDL cholesterol (mg/dl)	54.5(16.4)
LDL cholesterol (mg/dl)	94.1(27.9)
Triglycerides (mg/dl)	107.0(39.3)
Diabetes (%)	20.8
Drugs	
Acetylsalicylic acid (%)	33.3
ACE-inhibitors (%)	41.7
Angiontensin receptor blockers (%)	54.2
Calcium channels blockers (%)	75.0
Diuretics (%)	50.0
Mineralocorticoid receptor antagonists (%)	4.2
Doxazosin (%)	4.2
Statins (%)	62.5
Ezetimibe (%)	16.7
Metphormine (%)	8.3
CV Comorbidities	
TIA (%)	8.3
Stroke (%)	4.2
CCS (%)	4.2
Angina (%)	4.2
OSAS (%)	4.2
Pace-Maker implantation (%)	8.3
Non-CV Comorbidities	
Allergic asthma (%)	12.5
Hypothyroidism (%)	8.3
Anxiety (%)	4.2
COPD (%)	4.2
DLBCL in complete remission (%)	4.2
Cerebral ventricular hypertension (%)	4.2
Hepatic steatosis (%)	4.2
Secondary erythrocytosis (JAK2 negative) (%)	4.2
Thalassemia minor (%)	4.2

*BMI* Body mass index, *MET* Metabolic equivalent of task, *CKD* Chronic kidney disease, *HDL* High-density lipoprotein, *LDL* Low-density lipoprotein, *ACE* Angiotensin-converting enzyme, *CV* Cardiovascular, *TIA* Transitory ischemic attack, *CCS* Chronic coronary syndrome, *OSAS* Obstructive sleep apnea syndrome, *COPD* Chronic obstructive pulmonary disease, *DLBCL* Diffuse Large B Cell Lymphoma, *JAK2* Janus Kinase 2

### Procedures

All procedures were conducted in the cardiac rehabilitation outpatients department of the Niguarda Hospital, where participants attended a preliminary session to become familiar with the testing procedures. During this session, we collected a detailed medical history and performed a complete physical examination in all patients. Anthropometric characteristics (height and weight) were recorded using a mechanical scale to calculate body mass index (BMI). SBP and diastolic BP (DBP) values were taken with a semi-automatic sphygmomanometer. Additionally, carotid-to-femoral Pulse Wave Velocity (PWV_cf_) was measured using an automatic device, followed by the assessment of FMD in the brachial artery and single passive limb movement (sPLM) in the femoral artery with an ultrasound machine. Both PWV_cf_ and FMD were assessed unilaterally on the left side, while sPLM was performed on the right side. Upon completing the measurements, the position of the ultrasound probe was marked on a transparency sheet, along with notable skin landmarks (such as moles, scars, angiomas, etc.), to ensure accurate repositioning of the probe in subsequent evaluation sessions. The tests were administered in a consistent order across all evaluation sessions. Assessments were conducted at baseline (BASELINE) and after a 6-week control period without intervention (POST CTRL). This was followed by a 6-week intervention period involving PS training (POST STR). A final evaluation session was carried out after a 6-week follow-up period post-intervention (FOLLOW-UP). On evaluation days, participants were required to abstain from consuming ergogenic substances and antioxidant supplements for at least 48 h and to fast overnight. All sessions were conducted at the same time of day in a controlled environment with constant humidity and temperature to minimize any potential effects of circadian rhythms on biological factors (Morris et al. 2012).

### Laboratory analyses

An automatic analyser (Modular Analytics SWA, Roche Diagnostics, Monza, Italy) was used to assess biochemical parameters at baseline only, employing the following methods: total cholesterol was measured by enzymatic colorimetric assay (CHOD-PAP); HDL cholesterol by a third-generation enzymatic colorimetric assay without pretreatment; triglycerides by enzymatic colorimetric assay (GPO-PAP); and LDL cholesterol was calculated using the Friedewald equation. Creatinine levels were determined using an enzymatic method standardized against IDMS, and glucose was measured using an enzymatic colorimetric assay (GOD-PAP). Diabetes was defined as a fasting plasma glucose level > 126 mg/dL on two separate occasions or the use of antidiabetic medication (Maloberti et al. 2019).

### Passive stretching protocol

During the 6-week intervention period, participants followed a supervised PS protocol, performed 5 times per week, and did not engage in any other rehabilitation or training protocols that could have influenced the outcomes. Each session lasted 40 min and included 4 lower limb exercises (2 targeting the knee extensors and 2 targeting the plantar flexors), derived from a modified version of the PS protocol by Bisconti et al. (2020). The protocol was designed to minimize upper limb involvement and has previously been shown to improve knee joint range of motion after 6 weeks. Each training session took place in the cardiac rehabilitation section of Niguarda Hospital between 2:00 p.m. and 5:00 p.m. All exercises were assisted or supervised by a certified trainer to ensure correct execution, ensure the appropriate intensity (Cè et al. 2022), and help participants avoid co-contractions or unnecessary use of the upper limbs during stretching. Each exercise was performed bilaterally, one limb at a time, with the stretch held at the point of minimal discomfort for 45 s, followed by 15 s of rest between sets. Stretch intensity was progressively adjusted during each repetition and across sessions to consistently maintain the point of minimal discomfort. This intensity was selected to induce effective improvements in ROM while minimizing discomfort (Konrad et al. 2023). Five sets per limb were completed, resulting in a total of 10 min of stretching per exercise (Bisconti et al. 2020). Participants were instructed to avoid consuming ergogenic substances for at least 12 h prior to each session.

In the first exercise, participants lay prone on a padded table, with the hips and knees in a neutral position and the arms resting alongside the body. A certified trainer then extended the hip and flexed the participant’s knee, bringing the heel of the stretched limb toward the gluteus to achieve the desired stretching intensity. In the second exercise, participants stood upright, with support provided by the non-working limb and by a frontal support on which they could rest their forearms. Starting from a neutral hip and knee position, participants flexed the knee of the working limb while placing the dorsum of the foot on an adjustable posterior support. This allowed the heel to be brought closer to the gluteus to achieve the desired stretching intensity while minimizing co-contractions. In the third exercise, participants remained standing with the hips and knees in a neutral position. Balance was maintained using the same frontal support on which participants could rest their forearms. Participants then placed the sole of the foot on a step positioned in front of them and achieved maximal ankle dorsiflexion by shifting their center of mass forward until the appropriate stretching intensity was reached. In the final exercise, participants lay supine on a treatment table with the hips and knees in a neutral position and the arms resting alongside the body, while assisted by a certified trainer. The trainer flexed the participant’s hip and dorsiflexed the ankle while maintaining the knee in a neutral position, to achieve the desired stretching intensity. Participants who failed to attend at least 80% of the training sessions were excluded from the study, and new participants were recruited to replace any dropouts. During the study period, one participant withdrew due to health issues unrelated to the PS protocol. Attendance was 90.3% (SD 6.7%).

### Measurements and data analysis

#### Blood pressure and heart rate

BP and HR were selected as central parameters to evaluate cardiovascular responses at rest and to determine whether PS induced changes in systemic control of blood flow distribution. After the participants rested in a seated position for at least 5 min, BP measurements were taken by a trained physician using a manual mercury sphygmomanometer (OMRON Healthcare Europe, Hoofddorp, The Netherlands), with the cuff placed on the left arm, which was kept at heart level. Two measurements were taken, and the average was used for further analysis (Maloberti et al. 2019). Mean arterial pressure was calculated as (SBP + (DBPx2))/3. Resistant hypertension was defined as a SBP higher than 140 and/or a DBP higher 90 mmHg, despite at least 3 different anti-hypertensive drugs or a BP lower than the previous defined cut-off with 4 or more different anti-hypertensive drugs. After the participants rested supine on a padded bed approximately for 20 min in a quiet environment, heart rate (HR) was recorded using ECG for 5 min. The average value was then used for further analysis.

#### Pulse wave velocity (PWV_cf_)

PWV_cf_ was selected as a gold-standard, non-invasive index of central arterial stiffness, allowing us to evaluate whether passive stretching influenced large-artery mechanical properties. PWV_cf_ was measured by recording pressure pulse waves over the carotid and femoral arteries using an automatic device (Complior, Colson; Alam Medical, Paris, France). This device has been shown to be reliable and to provide recordings comparable to those obtained with applanation tonometry (Stea et al. 2014). The pressure pulse waveforms were simultaneously recorded at the two arterial sites on the left side, after measuring the distance between them with a rigid ruler. The transit time was obtained using the subtraction method, and the distance between the two recording sites was corrected by a factor of 0.8 to avoid overestimation of PWV_cf_ (Sugawara et al. 2008). The procedure was performed twice, and the average value was used for further analysis.

#### Flow mediated dilation (FMD)

**Fig. 1:**
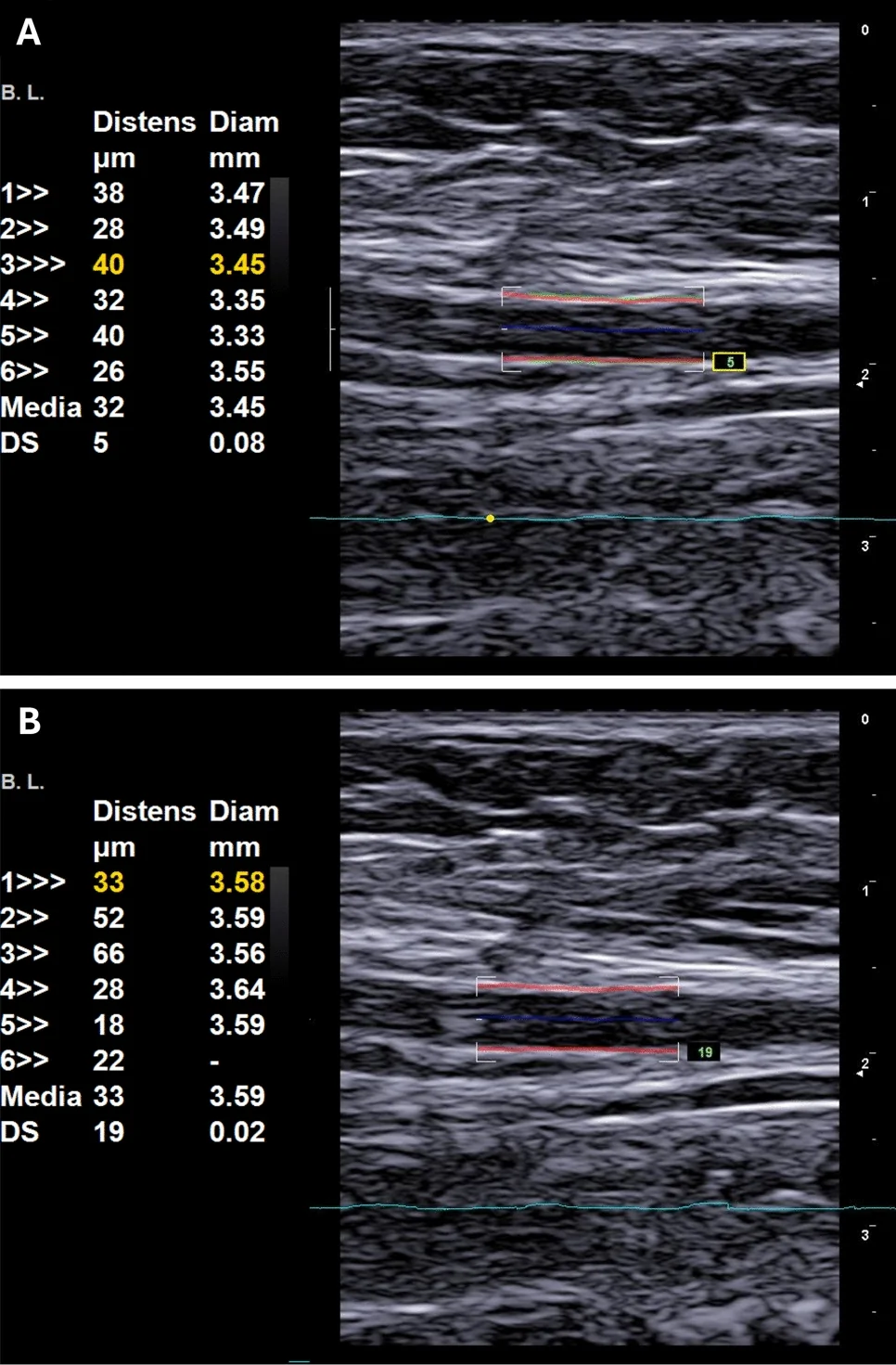
Automated edge-detection system. Flow-mediated dilation recording from a representative participant showing automated edge detection of brachial artery diameter before (**A**) and after (**B**) cuff occlusion, including details of the samples used to compute the mean diameter

FMD was used as a gold standard measure to assess endothelium-dependent vasodilation in the brachial artery, providing an indirect measure of systemic endothelial function and nitric oxide-mediated vascular responsiveness. This non-invasive test evaluates the vasodilatory response to reactive hyperaemia induced by increased shear stress and is widely used as an indicator of vasomotor function (Thijssen et al. 2011). Before FMD testing, the participants lay supine on a padded bed for approximately 20 min in a quiet environment to restore baseline cardiovascular values. FMD was performed on the left brachial artery by placing an arterial pressure cuff around the forearm, immediately distal to the olecranon process, following the recommended procedures (Bisconti et al. 2020). After baseline recording, the BP cuff was inflated to a supra-systolic pressure (250 mmHg) for 5 min to induce an ischemic stimulus. Recording resumed 30 s before cuff deflation and continued for an additional two minutes after deflation (Wray et al. 2013). During the procedure, a longitudinal image of the artery was recorded using a high-resolution ultrasound machine (ESAOTE MyLabα, Genova, Italy) in B mode with a 7.5 MHz linear array transducer. The probe was placed proximal to the bifurcation of the radial and ulnar arteries. Once a clear image was obtained, the probe was held stable with a stereotactic arm. Baseline and peak FMD diameters were assessed by measuring from one media-adventitia interface to the other at the end of diastole in the cardiac cycle, utilizing a real-time computerized edge detection system (Esaote, Genova, Italy) (Fig. 1). All data acquisition and analyses were performed by investigators blinded to the experimental conditions. FMD was quantified as the percentage increase in vessel diameter between the peak and baseline diameter. Baseline instantaneous blood flow was estimated using the baseline vessel diameter and the mean peak systolic blood velocity obtained from a 12-s ultrasound recording prior to cuff inflation, assuming a parabolic laminar flow profile. Instantaneous baseline blood flow (*Q̇*) was then calculated using the following formula:

$$\dot{Q}brac\left(\frac{ml}{min}\right)=V \times \pi \times {\left(vessel\frac{D}{2}\right)}^{2}\times 60$$

where *Q̇*_*brac*_ is the instantaneous baseline blood flow; *V* is the average corrected peak systolic velocity; *D* is the baseline diameter. Intra-session variability for flow-mediated dilation shows coefficients of variation of 8.5% (within-operator) and 9.4% (between-operator), respectively (Faverio et al. 2024).

#### Nitrate-mediated dilation (NMD)

NMD was assessed as a measure of endothelium-independent vasodilation, reflecting the smooth muscle’s ability to respond to an exogenous source of nitric oxide. This test allows evaluation of whether the vasodilatory capacity of the arterial media is preserved, ensuring that any changes observed in FMD are not confounded by impaired downstream vasodilatory function. NMD was performed in a randomly selected subset of 13 participants (66.2 ± 9.9 y; 29.4 ± 4.8 kg/m^2^). A Welch’s independent t-test showed no significant differences between included and excluded participants in age (*P* = 0.581), BMI (*P* = 0.163), baseline FMD (*P* = 0.122), or *Q̇*_*brac*_ (*P* = 0.685). After resting for at least 10 min, with the participants still in a supine position, 300 µg of nitroglycerin was administered via sublingual spray (Natispray®). Brachial artery diameter was measured at baseline and at 1, 3, and 5 min after drug administration. The maximum degree of dilation was used to calculate the percentage change from baseline.

#### Single passive limb movement (sPLM)

sPLM was used to assess endothelium-dependent vasodilation in the femoral artery, providing an indirect measure of local endothelial function and nitric oxide-mediated vascular responsiveness. Similarly to FMD, this test was used to evaluate the acute vascular response of the artery directly involved in the intervention, thereby capturing local adaptations in the vascular bed exposed to repeated stretching. The procedure was performed in the upright-seated position on the common femoral artery, distal to the inguinal ligament and proximal to the bifurcation of the deep and superficial femoral arteries, using a high-resolution ultrasound machine (ESAOTE MyLabα, Genova, Italy) in B-mode. Participants rested for 10 min prior to the collection of peripheral hemodynamic data (Bisconti et al. 2020). A 9 MHz linear array transducer was then used to record 30 s of baseline data, followed by an additional 59 s after a single passive knee flexion–extension movement lasting 1 s. This maneuver was performed by a member of the research team, who moved the participant’s leg through a 0–90° range of motion at 1 Hz and kept the leg extended for the remainder of the protocol. Instantaneous femoral blood flow (*Q̇*_*fem*_) was calculated at baseline as described for *Q̇*_*brac*_ measurement. Additionally, peak *Q̇*_*fem*_* was* evaluated considering the highest peak systolic velocity recorded in the 12 s following the single passive knee flexion–extension movement. Furthermore, ∆ *Q̇*_*fem*_ was calculated as the difference between peak *Q̇*_*fem*_ and baseline *Q̇*_*fem*_.

### Statistical analysis

Statistical analyses were performed using the open software jamovi, version 2.6.26. The Shapiro–Wilk test was used to assess the normality of data distribution. Continuous variables were described as mean and standard deviation (SD) while categorical ones were expressed as frequency (%).

Mauchly’s test evaluated the assumption of sphericity, and Greenhouse–Geisser corrections were applied when this assumption was violated. A one-way repeated-measures ANOVA was conducted to assess significant changes across time points (within-subject factor: *time*, 4 levels). When data were not normally distributed, Friedman’s test was used as a non-parametric alternative. Correlations between percentage differences in sPLM and BP variables were assessed with Pearson’s correlation coefficient. Statistical significance was set at *P* < 0.05, with Bonferroni or Durbin–Conover corrections applied for multiple comparisons when appropriate. Effect sizes for main effects and interactions were reported as partial eta squared (ηp^2^). The magnitude of pairwise comparisons was evaluated using Cohen’s *d* effect size (ES) and 95% confidence intervals. For non-normally distributed variables, rank biserial correlation was used to estimate effect sizes. Unless otherwise specified, data are presented as mean (SD).

## Results

### Population characteristics

Clinical characteristics of hypertensive patients are summarized in Table 1. 54.2% of the participants were male, with an age of 65.3 (SD 8.6) years, a BMI of 28.2 (SD 4.7) kg/m^2^ and 15.2 (SD 8.3) years of diagnosed hypertension. 45.8% were former smokers while only 4.2% were active smokers. Concerning antihypertensive drugs, patients were mainly treated with calcium channels blockers (75.0%), statins (62.5%) and angiotensin receptor blockers (54.2%). To note, 50.0% of the patients had resistant hypertension and 20.8% had diabetes.

### Blood pressure

**Fig. 2:**
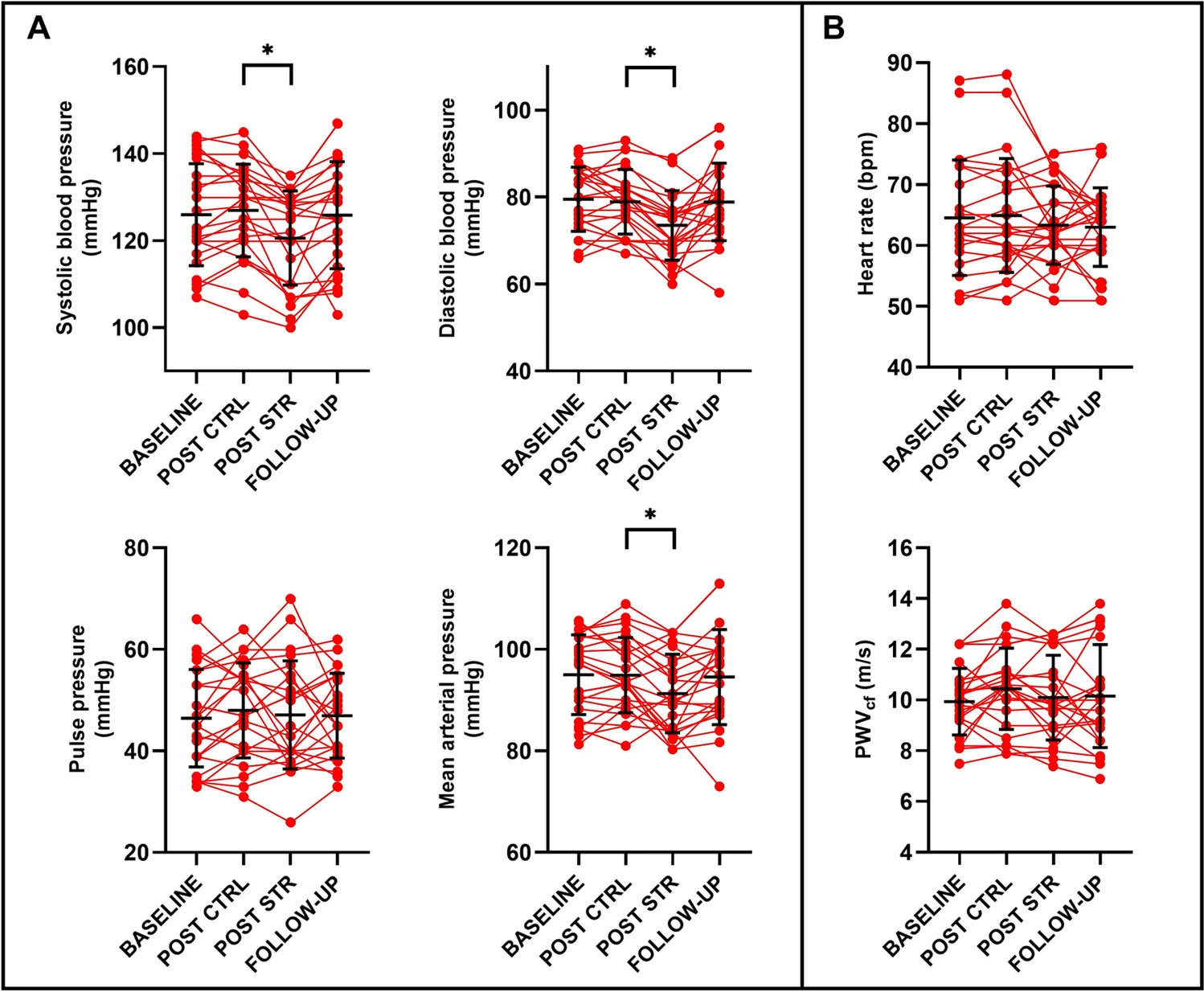
Central parameters. Individual and mean data for blood pressure parameters (**A**), heart rate and pulse wave velocity (**B**). *PWV*_*cf*_ carotid to femoral pulse wave velocity, *CTRL* control, *STR* stretching, * P < 0.05

BASELINE BP values were 126.0 (SD 11.7)/79.5 (SD 7.4) mmHg and its changes during the study are presented in Fig. 2, panel A. Friedman test disclosed a significant main effect for *time* in SBP (*χ*^*2*^ = 20.9, *P* < 0.001, *dF* = 3) which decreased from POST CTRL to POST STR by 6.3 (SD 6.0) mmHg [-4.9 (SD 4.6) %] (*P* < 0.001, *ES* = -0.99). Similarly, ANOVA disclosed a significant effect for *time* in DBP (*F*_*2.02*_ = 8.78, *P* = 0.001, *η*_*p*_^*2*^ = 0.28), and MAP (*F*_*1.88*_ = 3.59, *P* = 0.039, *η*_*p*_^*2*^ = 0.14) which decrease from POST CTRL to POST STR by 5.4 (SD 3.8) mmHg [-6.9 (SD 4.9) %] [*P* < 0.001, *ES* = -1.43 (-2.00 to -0.85)] and by 3.6 (SD 5.7) mmHg [-3.7 (SD 6.2) %] [*P* = 0.005, *ES* = -0.63 (-1.07 to -0.19)], respectively. No effect for *time* was retrieved in PP (*F*_*3*_ = 0.353, *P* = 0.787, *η*_*p*_^*2*^ = 0.02). In FOLLOW-UP, SBP, DBP and MAP returned to pre intervention values (*P* from 0.002 to 0.014 vs. POST STR; *P* from 0.423 to 0.980 vs. POST CTRL).

### HR and PWV_cf_

Changes in HR and PWV_cf_ during the intervention are reported on Fig. 2, panel B. No effect for *time* was retrieved in both HR (*F*_*1.41*_ = 0.77, *P* = 0.431, *η*_*p*_^*2*^ = 0.03) and PWV_cf_ (*F*_*3*_ = 1.50, *P* = 0.223, *η*_*p*_^*2*^ = 0.06).

### FMD and NMD

**Fig. 3:**
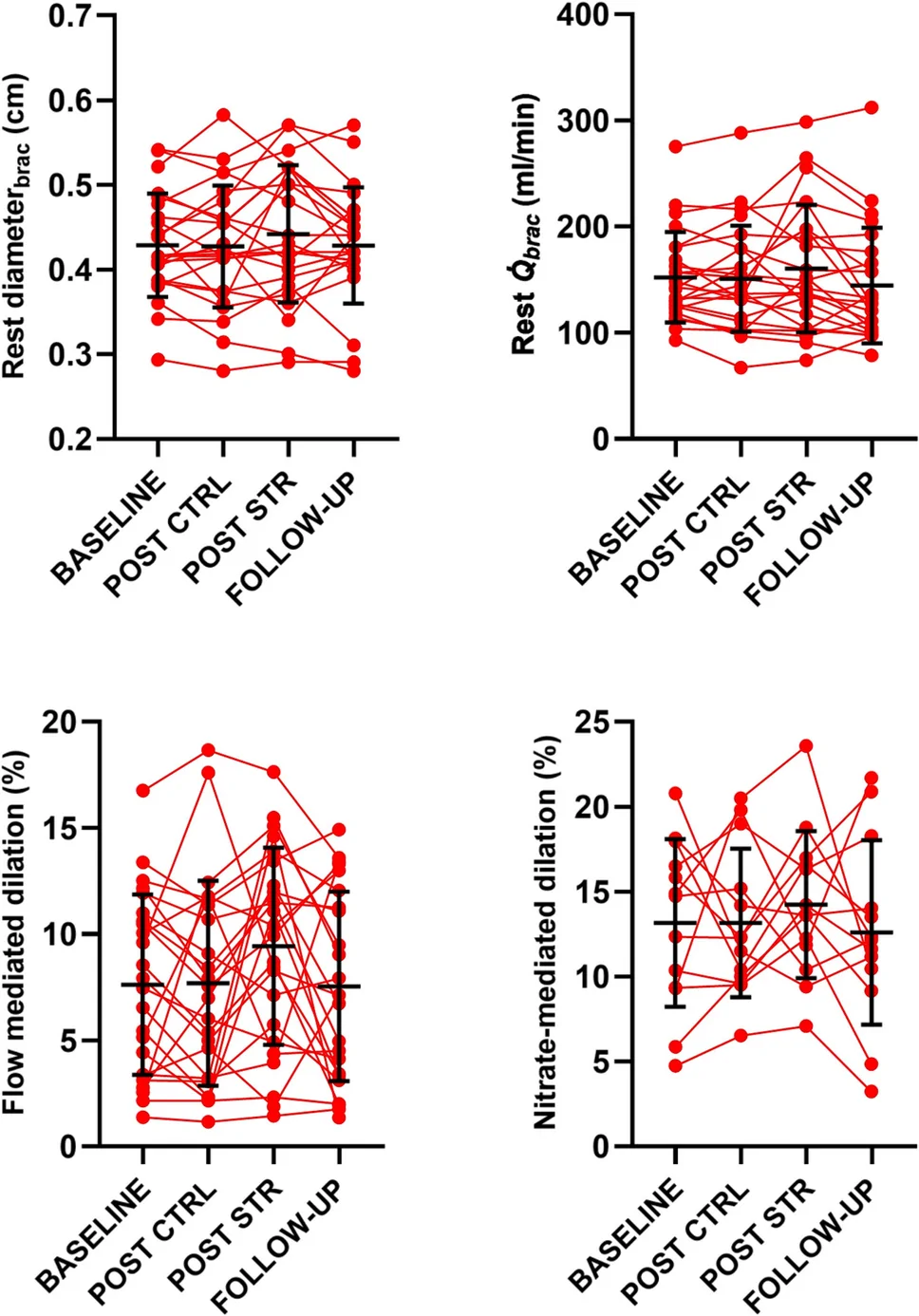
Systemic parameters. Individual and mean data for flow-mediated dilation and nitrate-mediated dilation variables performed on the brachial artery. *CTRL* control, *STR* stretching

Figure 3 presents the results of FMD and NMD performed over the brachial artery. No changes during the intervention period were found in baseline diameter (*F*_*3*_ = 1.17, *P* = 0.327, *η*_*p*_^*2*^ = 0.05), baseline *Q̇*_*brac*_ (*χ*^*2*^ = 4.31, *P* = 0.230, *dF* = 3), FMD (*F*_*3*_ = 1.96, *P* = 0.128, *η*_*p*_^*2*^ = 0.08) and NMD (*F*_*3*_ = 0.45, *P* = 0.722, *η*_*p*_^*2*^ = 0.04).

### sPLM

**Fig. 4:**
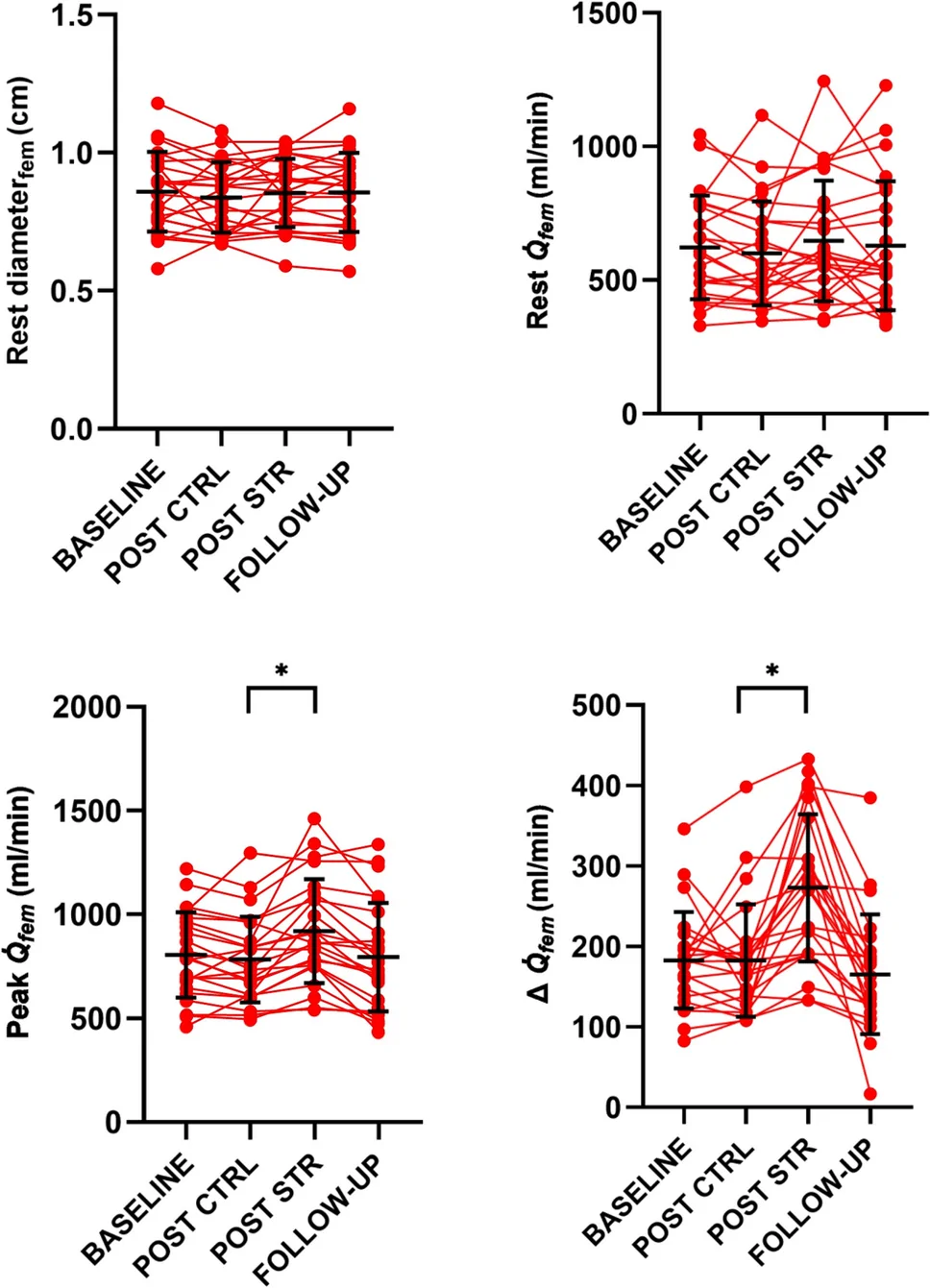
Local parameters. Individual and mean data for single passive limb variables performed on the femoral artery. *CTRL* control, *STR* stretching, *P < 0.001

Figure 4 shows the changes in sPLM parameters over the femoral artery. ANOVA disclosed a significant *time* interaction in peak *Q̇*_*fem*_ (*F*_*2.13*_ = 10.6, *P* < 0.001, *η*_*p*_^*2*^ = 0.32) and in ∆ *Q̇*_*fem*_ (*χ*^*2*^ = 39.8, *P* < 0.001, *dF* = 3). Peak *Q̇*_*fem*_ increased by 136.8 (SD 148.8) ml/min [+ 18.4 (SD 19.0) %] in POST STR [*P* < 0.001, *ES* = 0.92 (0.43 to 1.39)] while ∆ *Q̇*_*fem*_ increased by 90.3 (SD 86.4) ml/min [+ 27.1 (SD 21.7) %] compared to POST CTRL (*P* < 0.001, *ES* = 0.98). No changes after the intervention period were found in baseline diameter (*F*_*1.44*_ = 0.31, *P* = 0.664, *η*_*p*_^*2*^ = 0.01) and baseline *Q̇*_*fem*_ (*χ*^*2*^ = 3.78, *P* = 0.286, *dF* = 3). In FOLLOW-UP, peak *Q̇*_*fem*_ and ∆ *Q̇*_*fem*_ returned to pre intervention values (*P* < 0.001 vs. POST STR; *P* = 0.635 and 0.256 vs. POST CTRL).

### Correlations

**Fig. 5:**
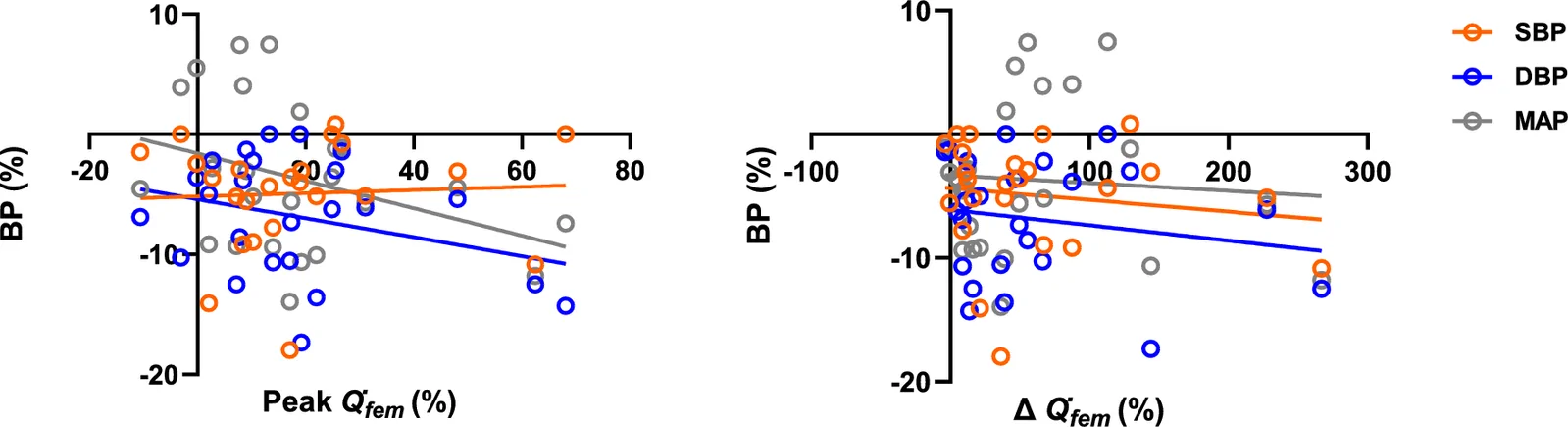
Correlations. Correlations between femoral artery blood flow (*Q̇*_*fem*_) and blood pressure (BP) variables, expressed as percentage changes from post-stretching to post-control. *SBP* systolic BP, *DBP* diastolic BP, *MAP* mean BP

The correlations between POST STR and POST CTRL percentage differences in *Q̇*_*fem*_ and BP variables are shown in Fig. 5. No significant correlations were found between Peak *Q̇*_*fem*_ and SBP (*r* = 0.06, *P* = 0.799), DBP (*r* = -0.31, *P* = 0.146) or MAP (*r* = -0.35, *P* = 0.091) and between ∆ *Q̇*_*fem*_ and SBP (*r* = -0.15, *P* = 0.496), DBP (*r* = -0.18, *P* = 0.403) or MAP (*r* = -0.07, *P* = 0.738).

## Discussion

This study aimed to evaluate whether a 6-week PS training protocol performed on the lower limbs could lower central arterial stiffness, improving both local and systemic vascular responsiveness leading to a reduced BP in hypertensive patients. Additionally, we assessed whether these improvements would be maintained six weeks after the cessation of the intervention. In partial agreement with our hypotheses, PS induced significant reductions in SBP, DBP, and MAP, alongside improvements in femoral artery vascular responsiveness at the end of the intervention. However, no changes were observed in PWV_cf_ or brachial artery vascular responsiveness. Importantly, all BP and vascular adaptations returned to baseline during follow-up, indicating a complete reversal of the training-induced effects. These findings suggest that PS can improve cardiovascular health in physically active hypertensive individuals, including those with resistant hypertension, and may represent a clinically relevant strategy to reduce cardiovascular risk and mortality (Ettehad et al. 2016).

### PS induced changes in blood pressure and central arterial stiffness

At the end of the PS training, we observed significant reductions in SBP (-6.3 mmHg), DBP (-5.4 mmHg), and MAP (-3.6 mmHg), comparable to those achieved with aerobic, resistance, and combined exercise training, despite PS being a potentially more tolerable intervention. No changes were observed in HR and PWV_cf_. Our results are partly in agreement with previous studies that investigated the effects of lower limb PS training on central arterial stiffness and BP in healthy individuals. Recently, it was demonstrated that a 4-week lower limb PS protocol reduced regional arterial stiffness in healthy middle-aged and older women, while no changes were observed in HR and central arterial stiffness (Higaki et al. 2022). However, the authors did not report any changes in BP values (Higaki et al. 2022). In contrast, in young and active subjects, central and regional stiffness, together with SBP and DBP, were reduced after 6 weeks when PS was performed bilaterally on the lower limbs (Bisconti et al. 2020). No studies, however, evaluated the effects of lower limb PS on BP and central stiffness in hypertensive patients, thus making a direct comparison difficult. Central and systemic adaptations would be promoted by the transient activation of the mechanoreflex during PS elongation phases, through stimulation of mechanoreceptors in the muscles directly involved in PS (Venturelli et al. 2017). Chronically, this would result in a remodulation of the sympathetic-vagal balance, along with a decrease in venous distending pressure, systemic arterial stiffness and BP (Kruse and Scheuermann 2017; Wong and Figueroa 2021). Our results suggest that in hypertensive subjects, six weeks of PS training are not sufficient to induce improvements attributed to systemic mechanisms, despite these individuals exhibiting an exaggerated responsiveness to mechanoreflex stimulation (Smith et al. 2015). The absence of significant correlations between percentage changes in *Q̇*_*fem*_ and BP parameters at POST STR suggests that the observed reductions in BP may be mediated by mechanisms other than the improvements in vascular responsiveness assessed in the present study. Although enhancements in vascular responsiveness often precede reductions in arterial stiffness and may partly explain the blood pressure reduction observed in this study (Kruse and Scheuermann 2017), other mechanisms should also be considered. In particular, changes in cardiac output and/or total peripheral resistance may have contributed to the response. In this context, the observed BP reduction is most plausibly explained by a decrease in total peripheral resistance rather than changes in cardiac output, as HR remained unchanged throughout the intervention. Additionally, the reduction in BP may have been influenced by subtle regional changes in the compliance of the central arterial tree that were not detected by PWV_cf_ measurements, particularly in hypertensive patients (Cuomo et al. 2017). In this regard, a larger sample size may have revealed significant changes in PWV_cf_ and FMD, as well as significant associations between vascular and hemodynamic adaptations, thereby providing a better explanation for the observed results. In the follow-up, the adaptations in SBP and DBP, and thus MAP, were not maintained, in line with previous findings (Shinno et al. 2017; Bisconti et al. 2020) implying the reversibility of the effects of stretching when the hemodynamic stimulus is not sustained.

### PS induced changes in vascular responsiveness

In partial agreement to our hypothesis, peak *Q̇*_*fem*_ and ∆ *Q̇*_*fem*_ improved in the femoral artery following 6 weeks of PS training, albeit these changes were not maintained in the follow-up, while no significant changes were observed in the brachial artery vascular responsiveness. The local PS-induced improvements observed here are consistent with previous studies, in which enhanced femoral artery vascular responsiveness was reported after 6 weeks in young healthy individuals (Bisconti et al. 2020). However, in our study, these changes did not persist at follow-up, suggesting that the adverse hypertensive condition on vascular responsiveness tends to prevail once the endothelial stimulus is withdrawn, especially in older adults (Higashi et al. 2012). Improvements in vascular responsiveness of the directly stretched arteries are attributed to the repeated exposure of the vessel lumen to shear forces (Venturelli et al. 2017; Kruse and Scheuermann 2017), which stimulate endothelial mechanotransduction signalling in smooth muscle cells, and ultimately lead to enhanced vasodilation, potentially due to increased NO bioavailability (Green et al. 2017). Previous research suggests that PS performed on the lower limbs can also enhance brachial artery vascular reactivity after just 8 weeks of training in young healthy individuals (Cè et al. 2022). FMD primarily assesses vascular responsiveness by measuring the vessel’s response to nitric oxide bioavailability (Wray et al. 2013); however, this parameter is also influenced by changes in sympathetic activity (Hijmering et al. 2002). In fact, non-local adaptations in vascular responsiveness are observed after 12 weeks of PS training (Bisconti et al. 2020), highlighting the potential role of systemic mechanisms in explaining the increase in FMD response. Nevertheless, the dysregulation of the autonomic nervous system that characterizes hypertensive patients (Mancia and Grassi 2014) may have hindered or limited the adaptations in brachial artery function, despite these individuals having a greater margin for improvement compared to younger, active subjects. Moreover, the relatively high NMD suggests the absence of impairments in vascular smooth muscle function (Maruhashi et al. 2020). Therefore, like what was discussed regarding central stiffness, an improvement in brachial artery vascular responsiveness may be promoted by local stimuli or by training protocols longer than 6 weeks, as vascular responsiveness and arterial compliance are closely related (Green et al. 2017). Furthermore, a larger sample size may also have increased statistical power to detect changes in brachial artery function and to better identify associations between vascular and hemodynamic adaptations.

### Study limitations

Several limitations should be acknowledged. Lifestyle factors such as physical activity, diet, body weight, and medication adherence were not strictly controlled and may have influenced the outcomes. In addition, the sequential study design introduces potential time-related confounders, including seasonal variation, learning effects, and behavioural adaptations. Moreover, because the intervention involved frequent supervised sessions whereas the control period did not, the absence of an attention-matched control condition prevents exclusion of potential placebo or supervision-related behavioural effects.

Mechanistic interpretation is also limited by the absence of direct assessments of endothelial function, limb-specific shear rate, regional arterial stiffness, and vascular resistance. Likewise, systemic biomarkers of autonomic and endothelial function were not evaluated. Autonomic regulation was inferred solely from heart rate, which does not fully capture sympathovagal balance; more comprehensive measures, such as heart rate variability or 24-h electrocardiographic monitoring, would have provided a more robust assessment. Similarly, BP was assessed using office measurements rather than ambulatory blood pressure monitoring, which may better reflect the true BP burden.

Furthermore, the advanced clinical profile of our participants, characterized by long-standing hypertension, polypharmacological treatment, and a high prevalence of resistant hypertension and comorbidities, may have limited vascular responsiveness and reduced the magnitude of detectable adaptations. In addition, the relatively small sample size may have limited the statistical power to detect changes in PWV_cf_, FMD, and their associations with BP outcomes.

Future studies should recruit larger cohorts, implement stricter control of lifestyle factors throughout the intervention, and incorporate more comprehensive assessments of autonomic function, local arterial stiffness, vascular resistance, and endothelial function to better elucidate the mechanisms underlying BP reductions following PS training. Moreover, different stretching modalities may induce greater or more rapid vascular adaptations in hypertensive individuals; therefore, future research should identify the most effective stretching strategies to optimize the dose–response relationship and maximize cardiovascular benefits.

## Conclusions

This study demonstrated that a 6-week lower-limb PS training protocol performed by hypertensive subjects: (a) improved femoral artery vascular responsiveness and reduced SBP, DBP, and MAP; (b) was insufficient to improve central arterial stiffness or systemic vascular responsiveness; (c) reduced BP through mechanisms not exclusively explained by changes in femoral vascular responsiveness; and (d) induced adaptations that were not maintained after PS cessation. Overall, these findings indicate a dissociation between local and systemic vascular adaptations, with benefits confined to the directly stretched vascular bed, although a larger sample size might have revealed systemic effects. Although improvements in femoral vascular responsiveness were accompanied by reductions in BP, the absence of a significant correlation between these changes suggests the involvement of additional physiological mechanisms not identified in the present study. Importantly, the magnitude of BP reduction was comparable to that reported for established exercise interventions and some pharmacological treatments, supporting PS as a highly tolerable and clinically relevant non-pharmacological strategy for BP management, even in individuals with resistant hypertension receiving stable antihypertensive therapy. Given the well-established association between BP reduction and lower cardiovascular risk, these findings may have important clinical implications for this population. However, the return of BP and vascular responsiveness to baseline levels during follow-up indicates that a continuous stretching stimulus is necessary to maintain these benefits, and represents a novel aspect of the present study by providing preliminary evidence on the time course and reversibility of these adaptations.

## Supplementary Information

Below is the link to the electronic supplementary material.

Supplementary file1 (XLSX 29 KB)

## Data Availability

Data collected and analysed during this study are presented as supplementary material.

## Abbreviations

BP: Blood pressure
DBP: Diastolic blood pressure
FMD: Flow-mediated dilation
HR: Heart rate
MAP: Mean arterial pressure
NMD: Nitrate-mediated dilation
PS: Passive static stretching
PWV_cf_: Carotid-to-femoral pulse wave velocity
SBP: Systolic blood pressure
sPLM: Single passive limb movement

## References

[CR1] Bisconti AV, Cè E, Longo S et al (2020) Evidence for improved systemic and local vascular function after long-term passive static stretching training of the musculoskeletal system. J Physiol 598:3645–3666. 10.1113/JP279866 32613634

[CR2] Bryant J, Cooper DJ, Peters DM, Cook MD (2023) The effects of static stretching intensity on range of motion and strength: a systematic review. JFMK 8:37. 10.3390/jfmk8020037 PMC10123604 37092369

[CR3] Cè E, Venturelli M, Bisconti AV et al (2022) Long-term passive leg stretch improves systemic vascular responsiveness as much as single-leg exercise training. Med Sci Sports Exerc 54:475–488. 10.1249/mss.0000000000002811 PMC10097495 34690287

[CR4] Cuomo F, Roccabianca S, Dillon-Murphy D et al (2017) Effects of age-associated regional changes in aortic stiffness on human hemodynamics revealed by computational modeling. PLoS ONE 12:e0173177. 10.1371/journal.pone.0173177 PMC5333881 28253335

[CR5] Dharmashankar K, Widlansky ME (2010) Vascular endothelial function and hypertension: insights and directions. Curr Hypertens Rep 12:448–455. 10.1007/s11906-010-0150-2 PMC2982873 20857237

[CR6] Edwards JJ, Deenmamode AHP, Griffiths M et al (2023) Exercise training and resting blood pressure: a large-scale pairwise and network meta-analysis of randomised controlled trials. Br J Sports Med 57:1317–1326. 10.1136/bjsports-2022-106503 37491419

[CR7] Ettehad D, Emdin CA, Kiran A et al (2016) Blood pressure lowering for prevention of cardiovascular disease and death: a systematic review and meta-analysis. Lancet 387:957–967. 10.1016/S0140-6736(15)01225-8 26724178

[CR8] Faverio P, Maloberti A, Rebora P et al (2024) Cardiovascular structural and functional parameters in idiopathic pulmonary fibrosis at disease diagnosis. High Blood Press Cardiovasc Prev 31:289–297. 10.1007/s40292-024-00638-0 PMC11161536 38739257

[CR9] Fitzgerald JD, Johnson L, Hire DG et al (2015) Association of objectively measured physical activity with cardiovascular risk in mobility-limited older adults. J Am Heart Assoc 4:e001288. 10.1161/JAHA.114.001288 PMC4345863 25696062

[CR10] Gabb G (2020) What is hypertension? Aust Prescr 43:108–109. 10.18773/austprescr.2020.025 PMC7450770 32921882

[CR11] Gimbrone MA, García-Cardeña G (2016) Endothelial cell dysfunction and the pathobiology of atherosclerosis. Circ Res 118:620–636. 10.1161/CIRCRESAHA.115.306301 PMC4762052 26892962

[CR12] Green DJ, Hopman MTE, Padilla J et al (2017) Vascular adaptation to exercise in humans: role of hemodynamic stimuli. Physiol Rev 97:495–528. 10.1152/physrev.00014.2016 PMC5539408 28151424

[CR13] Higaki Y, Fujie S, Yamato Y et al (2022) Four weeks of lower-limb static stretching reduces regional arterial stiffness in middle-aged and older women. Phys Act Nutr 26:022–027. 10.20463/pan.2022.0010 PMC9395250 35982626

[CR14] Higashi Y, Kihara Y, Noma K (2012) Endothelial dysfunction and hypertension in aging. Hypertens Res 35:1039–1047. 10.1038/hr.2012.138 22972557

[CR15] Hijmering ML, Stroes ESG, Olijhoek J et al (2002) Sympathetic activation markedly reduces endothelium-dependent, flow-mediated vasodilation. J Am Coll Cardiol 39:683–688. 10.1016/S0735-1097(01)01786-7 11849869

[CR16] Hotta K, Behnke BJ, Arjmandi B et al (2018) Daily muscle stretching enhances blood flow, endothelial function, capillarity, vascular volume and connectivity in aged skeletal muscle. J Physiol 596:1903–1917. 10.1113/JP275459 PMC5978284 29623692

[CR17] Hotta K, Batchelor WB, Graven J et al (2019) Daily passive muscle stretching improves flow-mediated dilation of popliteal artery and 6-minute walk test in elderly patients with stable symptomatic peripheral artery disease. Cardiovasc Revasc Med 20:642–648. 10.1016/j.carrev.2019.05.003 PMC6698217 31171470

[CR18] Humphrey JD, Harrison DG, Figueroa CA et al (2016) Central artery stiffness in hypertension and aging: a problem with cause and consequence. Circ Res 118:379–381. 10.1161/CIRCRESAHA.115.307722 PMC4745997 26846637

[CR19] Kato M, Green FN, Hotta K et al (2020) The efficacy of stretching exercises on arterial stiffness in middle-aged and older adults: a meta-analysis of randomized and non-randomized controlled trials. Int J Environ Res Public Health 17:1–15. 10.3390/ijerph17165643 PMC7460052 32764418

[CR20] Kim H-L (2023) Arterial stiffness and hypertension. Clin Hypertens 29:31. 10.1186/s40885-023-00258-1 PMC10691097 38037153

[CR21] Kim H-L, Kim S-H (2019) Pulse wave velocity in atherosclerosis. Front Cardiovasc Med. 10.3389/fcvm.2019.00041 PMC6465321 31024934

[CR22] Ko J, Deprez D, Shaw K et al (2020) Stretching is superior to brisk walking for reducing blood pressure in people with high-normal blood pressure or stage I hypertension. J Phys Act Health 18:21–28. 10.1123/jpah.2020-0365 33338988

[CR23] Konrad A, Alizadeh S, Daneshjoo A et al (2023) Chronic effects of stretching on range of motion with consideration of potential moderating variables: a systematic review with meta-analysis. J Sport Health Sci. 10.1016/j.jshs.2023.06.002 PMC10980866 37301370

[CR24] Kruse NT, Scheuermann BW (2017) Cardiovascular responses to skeletal muscle stretching: “stretching” the truth or a new exercise paradigm for cardiovascular medicine? Sports Med 47:2507–2520. 10.1007/s40279-017-0768-1 28780647

[CR25] Kruse NT, Silette CR, Scheuermann BW (2016) Influence of passive stretch on muscle blood flow, oxygenation and central cardiovascular responses in healthy young males. Am J Physiol Heart Circ Physiol 310:H1210–H1221. 10.1152/ajpheart.00732.2015 26945077

[CR26] Lim PO, MacFadyen RJ, Clarkson PBM, MacDonald TM (1996) Impaired exercise tolerance in hypertensive patients. Ann Intern Med 124:41–55. 10.7326/0003-4819-124-1_Part_1-199601010-00008 7503477

[CR27] Maloberti A, Rebora P, Andreano A et al (2019) Pulse wave velocity progression over a medium-term follow-up in hypertensives: focus on uric acid. J Clin Hypertens (Greenwich) 21:975–983. 10.1111/jch.13603 PMC8030372 31222917

[CR28] Mancia G, Grassi G (2014) The autonomic nervous system and hypertension. Circ Res 114:1804–1814. 10.1161/CIRCRESAHA.114.302524 24855203

[CR29] Maruhashi T, Kajikawa M, Kishimoto S et al (2020) Diagnostic criteria of flow-mediated vasodilation for normal endothelial function and nitroglycerin-induced vasodilation for normal vascular smooth muscle function of the brachial artery. J Am Heart Assoc 9:e013915. 10.1161/JAHA.119.013915 PMC7033833 31910779

[CR30] Moiz A, Zolotarova T, Eisenberg MJ (2024) Outpatient management of essential hypertension: a review based on the latest clinical guidelines. Ann Med 56:2338242. 10.1080/07853890.2024.2338242 PMC11011233 38604225

[CR31] Morris CJ, Yang JN, Scheer FAJL (2012) The impact of the circadian timing system on cardiovascular and metabolic function. Prog Brain Res 199:337–358. 10.1016/B978-0-444-59427-3.00019-8 PMC3704149 22877674

[CR32] Nishiwaki M, Yonemura H, Kurobe K, Matsumoto N (2015) Four weeks of regular static stretching reduces arterial stiffness in middle-aged men. SpringerPlus. 10.1186/s40064-015-1337-4 PMC4583555 26435901

[CR33] Oparil S, Acelajado MC, Bakris GL et al (2018) Hypertension. Nat Rev Dis Primers 4:18014. 10.1038/nrdp.2018.14 PMC6477925 29565029

[CR34] Sandoo A, van Zanten JJCSV, Metsios GS et al (2010) The endothelium and its role in regulating vascular tone. Open Cardiovasc Med J 4:302–312. 10.2174/1874192401004010302 PMC3040999 21339899

[CR35] Saz-Lara A, Bruno RM, Cavero-Redondo I et al (2022) Association between arterial stiffness and blood pressure progression with incident hypertension: a systematic review and meta-analysis. Front Cardiovasc Med 9:798934. 10.3389/fcvm.2022.798934 PMC8873377 35224042

[CR36] Shinno H, Kurose S, Yamanaka Y et al (2017) Evaluation of a static stretching intervention on vascular endothelial function and arterial stiffness. Eur J Sport Sci 17:586–592. 10.1080/17461391.2017.1284267 28287021

[CR37] Smith SA, Leal AK, Murphy MN et al (2015) Muscle mechanoreflex overactivity in hypertension: a role for centrally-derived nitric oxide. Auton Neurosci 188:58–63. 10.1016/j.autneu.2014.12.004 PMC4365425 25630887

[CR38] Stea F, Bozec E, Millasseau S et al (2014) Comparison of the Complior Analyse device with Sphygmocor and Complior SP for pulse wave velocity and central pressure assessment. J Hypertens 32:873. 10.1097/HJH.0000000000000091 24509122

[CR39] Sugawara J, Hayashi K, Yokoi T, Tanaka H (2008) Age-associated elongation of the ascending aorta in adults. JACC Cardiovasc Imaging 1:739–748. 10.1016/j.jcmg.2008.06.010 19356510

[CR40] Themistocleous I-C, Michael C, Hadjisavvas S et al (2026) Effects of different types of stretching on hypertension: a systematic review with exploratory meta-analysis. J Funct Morphol Kinesiol 11:164. 10.3390/jfmk11020164 PMC13108135 42029532

[CR41] Thijssen DHJ, Black MA, Pyke KE et al (2011) Assessment of flow-mediated dilation in humans: a methodological and physiological guideline. Am J Physiol Heart Circ Physiol 300:H2–H12. 10.1152/ajpheart.00471.2010 PMC3023245 20952670

[CR42] Thomas E, Bellafiore M, Gentile A et al (2021) Cardiovascular responses to muscle stretching: a systematic review and meta-analysis. Int J Sports Med 42:481–493. 10.1055/a-1312-7131 33440445

[CR43] Venturelli M, Cè E, Limonta E et al (2017) Central and peripheral responses to static and dynamic stretch of skeletal muscle: mechano- and metaboreflex implications. J Appl Physiol 122:112–120. 10.1152/japplphysiol.00721.2016 27856718

[CR44] Venturelli M, Rampichini S, Coratella G et al (2019) Heart and musculoskeletal hemodynamic responses to repetitive bouts of quadriceps static stretching. J Appl Physiol 127:376–384. 10.1152/japplphysiol.00823.2018 31161884

[CR45] Widlansky ME, Gokce N, Keaney JF, Vita JA (2003) The clinical implications of endothelial dysfunction. J Am Coll Cardiol 42:1149–1160. 10.1016/S0735-1097(03)00994-X 14522472

[CR46] Wong A, Figueroa A (2014) Eight weeks of stretching training reduces aortic wave reflection magnitude and blood pressure in obese postmenopausal women. J Hum Hypertens 28:246–250. 10.1038/jhh.2013.98 24132138

[CR47] Wong A, Figueroa A (2021) Effects of acute stretching exercise and training on heart rate variability: a review. J Strength Cond Res 35:1459–1466. 10.1519/jsc.0000000000003084 30789584

[CR48] Wray DW, Witman MAH, Ives SJ et al (2013) Does brachial artery flow-mediated vasodilation provide a bioassay for NO? Hypertension 62:345–351. 10.1161/HYPERTENSIONAHA.113.01578 PMC3775568 23774225

[CR49] Yamada E, Sakai S, Uchiyama M et al (2022) Compliance of static stretching and the effect on blood pressure and arteriosclerosis index in hypertensive patients. Clin Pract 12:306–317. 10.3390/clinpract12030036 PMC9149945 35645313

